# Development of prognostic models for advanced multiple hepatocellular carcinoma based on Cox regression, deep learning and machine learning algorithms

**DOI:** 10.3389/fmed.2024.1452188

**Published:** 2024-09-27

**Authors:** Jie Shen, Yu Zhou, Junpeng Pei, Dashuai Yang, Kailiang Zhao, Youming Ding

**Affiliations:** ^1^Department of Hepatobiliary Surgery, Renmin Hospital of Wuhan University, Wuhan, China; ^2^Department of Hepatobiliary Surgery, 521 Hospital of Norinco Group, Xi’an, China

**Keywords:** advanced multiple hepatocellular carcinoma, prognosis, machine learning, deep learning, gradient boosted machine

## Abstract

**Background:**

Most patients with multiple hepatocellular carcinoma (MHCC) are at advanced stage once diagnosed, so that clinical treatment and decision-making are quite tricky. The AJCC-TNM system cannot accurately determine prognosis, our study aimed to identify prognostic factors for MHCC and to develop a prognostic model to quantify the risk and survival probability of patients.

**Methods:**

Eligible patients with HCC were obtained from the Surveillance, Epidemiology, and End Results (SEER) database, and then prognostic models were built using Cox regression, machine learning (ML), and deep learning (DL) algorithms. The model’s performance was evaluated using C-index, receiver operating characteristic curve, Brier score and decision curve analysis, respectively, and the best model was interpreted using SHapley additive explanations (SHAP) interpretability technique.

**Results:**

A total of eight variables were included in the follow-up study, our analysis identified that the gradient boosted machine (GBM) model was the best prognostic model for advanced MHCC. In particular, the GBM model in the training cohort had a C-index of 0.73, a Brier score of 0.124, with area under the curve (AUC) values above 0.78 at the first, third, and fifth year. Importantly, the model also performed well in test cohort. The Kaplan–Meier (K-M) survival analysis demonstrated that the newly developed risk stratification system could well differentiate the prognosis of patients.

**Conclusion:**

Of the ML models, GBM model could predict the prognosis of advanced MHCC patients most accurately.

## 1 Introduction

Hepatocellular carcinoma (HCC), the sixth most common cancer in the world, has an insidious onset, rapid progression and poor prognosis, making it more difficult to treat (1). Accurately assessing the prognosis of HCC patients may provide clinicians reference values to develop more effective treatment plans. AJCC-TNM and Barcelona Clinic Liver Cancer (BCLC) staging are the most commonly used staging systems for HCC, but they are unable to take into account the effects of treatment, age, and other important factors, thus, they seem to have poor accuracy (2). Recently, a large number of scholars have used nomograms to study cancer (3, 4), which were built based on multifactorial Cox regression analyses with fixed weights assigned, and the accuracy is sometimes unsatisfactory (5). Machine Learning (ML) enables computers to learn from large-scale, disparate healthcare data and then make decisions or predictions without being explicitly programmed. ML models offer considerable advantages over traditional statistical models for tasks such as diagnosis, classification and survival prediction (6, 7). DL is a branch of ML that uses a ML technique called artificial neural networks to extract patterns and make predictions from large datasets, and is particularly well suited to solving complex computational problems (8).

MHCC is classified into two types, one is intrahepatic metastasis, which is the result of intrahepatic metastasis of solitary tumor nodule, and the other is multicentric origin, which is the primary HCC (9). There are few studies on the prognosis of MHCC, making treatment more difficult (10). Previous studies have revealed that tumor size, Alpha-Fetal Protein (AFP) level, surgical treatment, microvascular invasion and hepatic functional status were important risk factors affecting patients’ recurrence or Overall Survival (OS) (11–13). As for surgery, although many studies have consistently shown that surgery is beneficial in MHCC (11, 14, 15), there are no studies that indicate whether patients with advanced MHCC can benefit from it.

The aim of this study is to construct prognostic models based on Cox regression, ML and DL algorithms using a large dataset from the SEER database, to predict the prognosis of patients with advanced MHCC, thus helping clinicians to optimize their decisions.

## 2 Methods

### 2.1 Selection of patients and study variables

**Figure 1:**
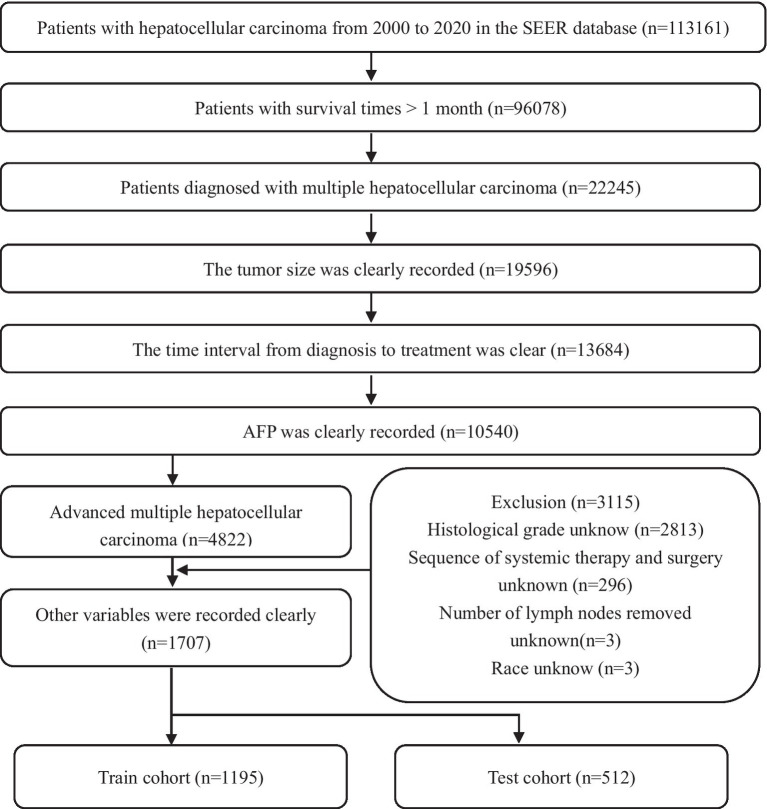
Flow chart of patients’ selection in the training and test cohorts from the SEER database.

Data on patients, who were diagnosed with HCC between 2000 and 2020, were obtained with SEER*Stat software (version 8.4.2). The SEER database is publicly accessible and does not require approval by the ethics institutional review board. External validation data were obtained from Renmin Hospital of Wuhan University. Variables included in the study were age, sex, race, tumor size, tumor primary site and regional lymph surgery information, months from diagnosis to treatment, AJCC-TNM stage, histological grade, radiotherapy, chemotherapy, AFP, sequence of systemic therapy and surgery, number and sequence of malignant tumors, a total of 15 variables. Patient inclusion criteria: (1) Patients diagnosed with HCC between 2000 and 2020 (histologic type International Classification of Disease for Oncology third edition = 8,170–8,175) (ICD-O3); (2) CS extension records as multiple nodules; (3) TNM stage was stage III or IV. The exclusion criteria are as follows: (1) Data missing or not clearly recorded, grouping disputed data; (2) Survival time is not recorded or less than 1 month; (3) A patient has two or more medical records, the last one shall prevail. The detailed selection process was shown in Figure 1.

### 2.2 Variable selection and construction of prognostic models

We randomly divided 1707 advanced MHCC patients into a training cohort and test cohort in a 7:3 ratio. Univariate and multivariate Cox were successively used to screen variables with prognostic significance, that is, Variables with hazard ratio (HR) more or less than 1 and statistically significant were retained. Use R software (version 4.2.1), open-source Python library scikit-survival (version 0.21.0) and PyTorch (Python version 3.11.4) to build prediction models (16).

### 2.3 Evaluation and selection of the best prediction model

Calculating C-index and Brier score to assess the accuracy of model prediction, receiver operating characteristic (ROC) curves and decision curve analysis (DCA) curves for the 1st, 3rd, and 5th year were then continued to be plotted to compare the accuracy of the models and potential clinical benefit (17). We determined the best cut off value for risk grouping by X-tile software, then K-M curves were used to compare the differences in OS of advanced MHCC patients in different risk stratification groups.

### 2.4 Interpretation of GBM model

The explanation of the model was divided into two parts: SHAP plot and the prediction website based on JAVA. SHAP is a model interpretation package developed in Python, for each prediction sample, the SHAP value is assigned to each feature. The larger the absolute value of SHAP, the greater the influence of the feature, and the sign of the value indicates whether the feature has a positive or negative effect on the result (18, 19). In order to better present the results and make it easier for the reader to use the model, an interactive website was established. By entering the required clinical information, 1-, 3-, and 5-year survival probability and risk score can be automatically calculated.

### 2.5 Statistical analysis

All statistical analyses were performed by R software (version 4.2.1.) and Python (version 3.11.4.) The “survival” package and “survminer” package were used for univariate and multivariate Cox regression analysis, forest mapping. Hazard ratio (HR) > 1 indicates that the factor is a risk factor, while HR < 1 indicates it is a protective factor. The “rms” package was used to draw the nomogram. Survival distributions were compared using the log-rank test. All tests were two-sided and *p* values less than 0.05 were considered statistically significant.

## 3 Results

### 3.1 Baseline characteristics in the training and test cohorts

A total of 1707 advanced MHCC patients were enrolled in our study, including 1,195 (70%) in the training cohort and 512 (30%) in the internal test cohort, the information for the external cohort can be obtained from Supplement Sheet 1. Most of these patients only had HCC, and their histological grading was in grades I to III, with a predominance of grade II. The vast majority of patients were at stage IIIA in the AJCC-TNM staging, i.e., there were multiple lesions in liver and any one of the lesions was more than 5 cm in size without lymph node or major vascular invasion. Because of this, the vast majority of tumor size was greater than 5 cm, but dimensions greater than 10 cm were rare. AFP is often considered as a marker for HCC, although the sensitivity and specificity are not satisfactory. In this study, AFP was abnormal in more than 70% of patients with advanced MHCC. In terms of treatment, not many patients were treated immediately after being diagnosed, they were more likely to choose to receive treatment after one to 2 months, and, of course, more than 10% of patients still went for treatment in the fourth month or later. There is a gap in research regarding surgery in advanced MHCC. Nearly 70% of the patients in this study did not undergo surgical treatment, still more than 20% underwent partial hepatectomy, in addition, almost all patients did not undergo lymph node dissection. Table 1 detailed the baseline information of the patients with advanced MHCC in this study.

**Table 1:** Demographic and clinical characteristics of patients with advanced MHCC.

Variable	Overall		Train cohort		Test cohort	
	Sample size	Percentage	Sample size	Percentage	Sample size	Percentage
Total	1707		1,195		512	
Histological grade^a^						
I	488	28.59%	331	27.70%	157	30.66%
II	791	46.34%	557	46.61%	234	45.71%
III	400	23.43%	287	24.02%	113	22.07%
IV	28	1.64%	20	1.67%	8	1.56%
TNM stage						
IIIA	1,236	72.41%	860	71.97%	376	73.43%
IIIB	8	0.47%	6	0.50%	2	0.39%
IIIC	117	6.85%	80	6.69%	37	7.23%
IV	346	20.27%	249	20.84%	97	18.95%
Diagnosis to treat^b^						
Zero	272	15.93%	197	16.49%	75	14.65%
One	576	33.74%	397	33.22%	179	34.95%
Two	408	23.90%	294	24.60%	114	22.27%
Three	228	13.36%	159	13.31%	69	13.48%
4 or more	223	13.07%	148	12.38%	75	14.65%
Primary site surgery						
No surgery	1,162	68.07%	811	67.87%	351	68.55%
Local tumor destruction	103	6.03%	80	6.69%	23	4.50%
Partial hepatectomy	394	23.09%	270	22.59%	124	24.22%
Liver transplantation	48	2.81%	34	2.85%	14	2.73%
Tumor size						
< 5 cm	133	7.79%	95	7.95%	38	7.42%
5 ~ 10 cm	1,066	62.45%	744	62.26%	322	62.89%
> 10 cm	508	29.76%	356	29.79%	152	29.69%
Lymph surgery						
No	1,591	93.20%	1,108	92.72%	483	94.33%
Biopsy	9	0.53%	8	0.67%	1	0.20%
Yes	107	6.27%	79	6.61%	28	5.47%
AFP						
Negative	437	25.60%	292	24.44%	145	28.32%
Borderline	7	0.41%	5	0.42%	2	0.39%
Positive	1,263	73.99%	898	75.14%	365	71.29%
Sequence number^c^						
One primary only	1,388	81.31%	971	81.26%	417	81.45%
1st of 2 or more	58	3.40%	42	3.51%	16	3.13%
Not 1st of 2 or more	261	15.29%	182	15.23%	79	15.42%

a “Grade” refers to histological grade. In pathological reports, highly differentiated ICC corresponds to “Grade I,” moderately differentiated ICC corresponds to “Grade II,” poorly differentiated ICC corresponds to “Grade III,” and undifferentiated ICC corresponds to “Grade IV”.

b “Diagnosis to Treat” refers to time interval from diagnosis to treatment.

c “Sequence number” refers to the order in which the cancers in a patient’s lifetime compared to ICC. “One primary only” means that the patient has only ICC in his or her lifetime. “1st of 2 or more” “1st of 2 or more” means that the ICC is the patient’s first malignant tumor, but later developed other tumors. “not 1st primary” means that the patient had other tumors prior to ICC.

### 3.2 Screening for statistically significant prognostic factors

**Figure 2:**
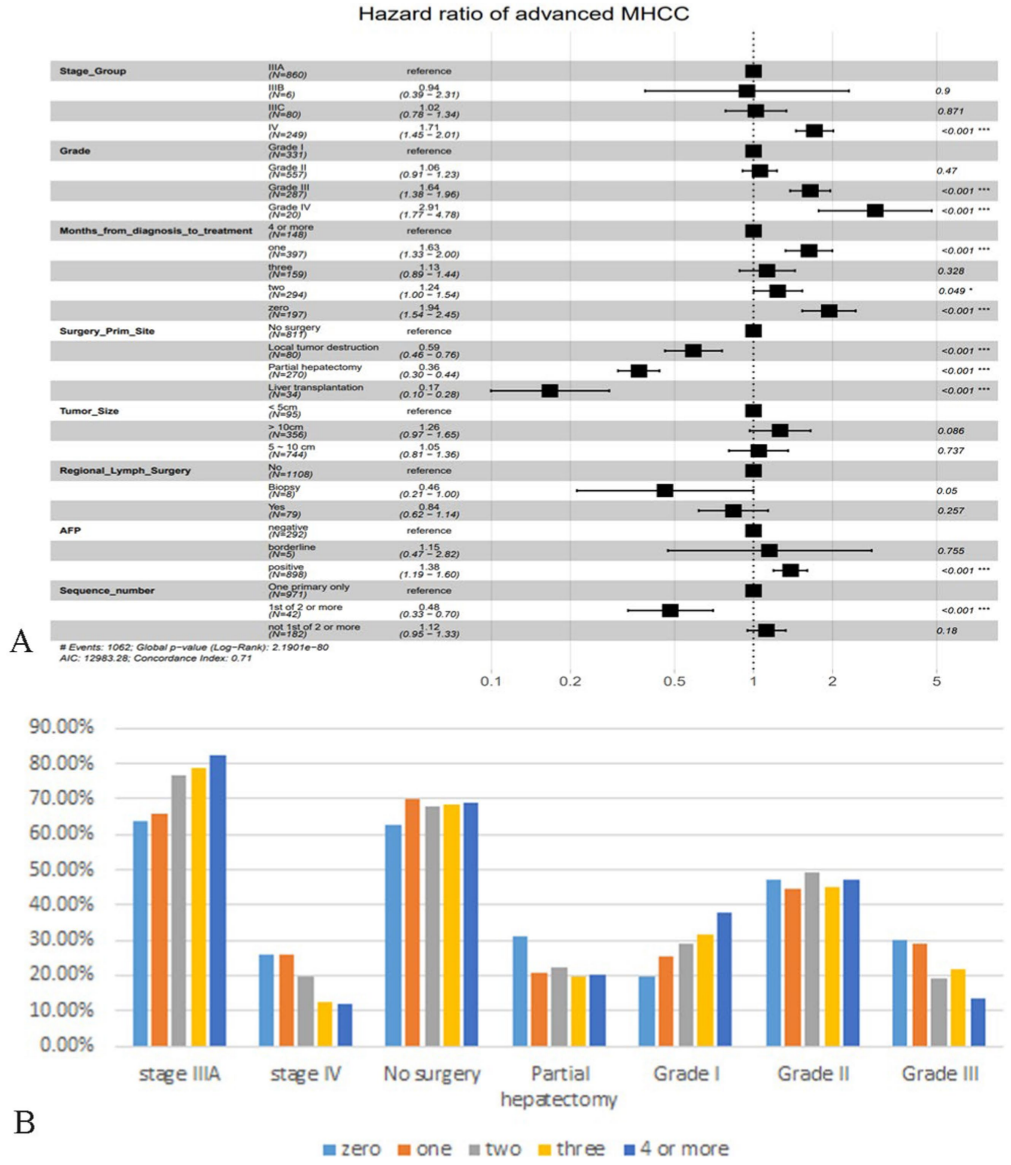
Demonstration of multivariate Cox regression analysis and analysis of patients in different months from diagnosis to treatment. **(A)** Forest plot based on multivariate Cox regression analysis. **(B)** Bar plot of important features of advanced MHCC patients in different months from diagnosis to treatment. The vertical coordinate is the percentage of the feature subgroup in the group.

A total of 15 variables were included in the study, after univariate Cox analysis, as shown in Supplementary Table S1, four variables: age, race, number of malignant tumors and radiotherapy were excluded. A multivariate analysis was conducted immediately afterward, the results showed that TNM stage, histological grade, months from diagnosis to treatment, primary site surgery, tumor size, regional lymph surgery, AFP and sequence of malignant tumors were independent prognostic factors for patients with advanced MHCC, therefore, a total of 8 prognostic factors with statistical significance (Figure 2A).

As shown in the figure, it is clear that patients with AJCC-TNM staging at stage IV had a higher risk of death than those at stage IIIA, and stage IIIB may be a false positive because the proportion of patients was too small. The results regarding histologic grade were consistent with popular knowledge that the lower the degree of differentiation, the correspondingly lower the OS of the patient. Surprisingly, “time interval from diagnosis to treatment” was not the factor that patients who were treated immediately had a better prognosis, patients who were treated immediately after diagnosis or who received treatment a month later had a significantly higher risk of death than those who were delayed for 4 months or more. We tried to analyze whether it was influenced by other factors, selecting some of the important ones. Figure 2B showed that “zero” or “one” group had a significantly lower proportion of patients in stage IIIA than the “4 or more” group, and a significantly higher proportion in stage IV (Figure 2B). The same trend was observed in the factor histological grade, so they may have influenced the significance of the factor “months from diagnosis to treatment” on prognosis. As for surgery for tumor lesions, liver transplantation (LT) remained the best treatment modality, greatly reducing the risk of death, and failure to undergo surgery appeared to be the highest risk. In this study, we did not find significant variability between subgroups of tumor size and subgroups of regional lymph surgery. The risk of death was significantly higher in the AFP-positive group than in the negative group, and surprisingly, the risk of death in MHCC patients who recurred other primary tumors was instead lower than that of MHCC only, which we discussed in the Discussion section.

### 3.3 Evaluation and comparison of prognostic models

**Figure 3:**
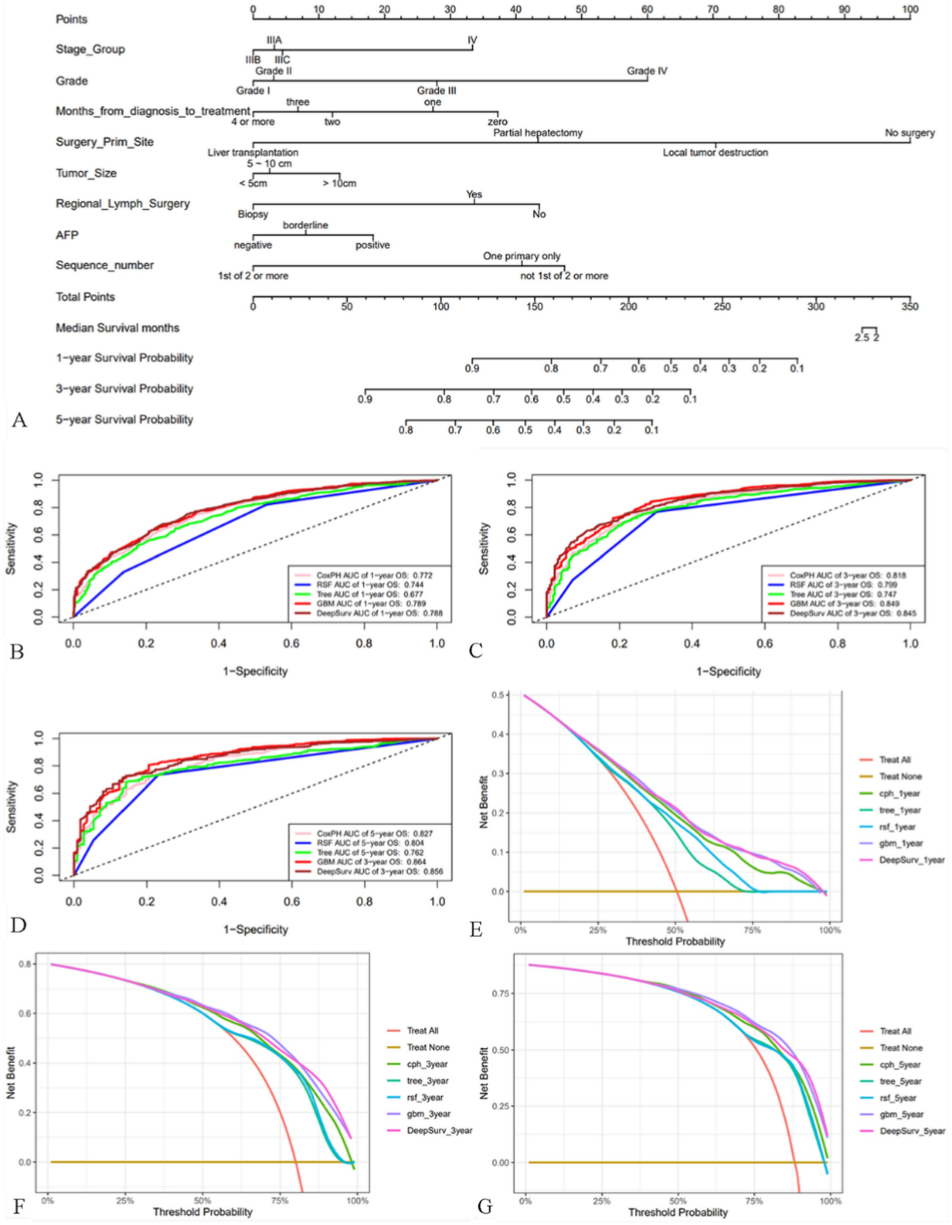
Nomogram of patients with advanced MHCC and evaluation of the performance of the five models. **(A)** Nomogram of patients with advanced MHCC. **(B–D)** ROC curves for prognostic models predicting 1-, 3-, and 5-year OS in the training cohort. (**E–G**) DCA curves of prognostic models for 1-year, 3-year, and 5-year OS prediction in the training cohort.

Based on the training cohort, we first constructed a nomogram model using R software (Figure 3A), which is a visualization of multivariate Cox regression analysis with the same performance as Cox proportional hazards (CPH) model (20). Nomogram is convenient to use, but it is not hard to notice from Supplementary Table S2 that although its Brier score is not high, its C-index is 0.71, which is unsatisfactory. So based on ML and DL algorithms, we constructed CPH, survival tree, random survival forest (RSF), GBM and DeepSurv model, a total of five models, and optimized the parameters of models with five-fold cross-validation (Supplementary Table S3; Supplement Sheet 1). We first calculated their C-index, Brier score to evaluate the models as shown in Supplementary Table S2. Obviously, the GBM model performed the best with a high C-index of 0.73 and a low Brier score of 0.111. We then plotted the ROC curves for the 1st, 3rd, and 5th year of the five models (Figures 3B–D), and we can note that the GBM model always had the highest area under the curve (AUC) values, followed by the DeepSurv model. Interestingly, the AUC values gradually increased with time, suggesting that the GBM model is more accurate in predicting long-term prognosis. DCA curves showed (Figures 3E–G) that using our models to guide treatment can bring benefits to patients, with the GBM and DeepSurv models leading to more benefits for patients with advanced MHCC. In summary, it is not difficult to conclude that the GBM model outperformed the other models, so we selected the GBM model for subsequent evaluation and research.

### 3.4 Validation of GBM performance and development of a risk stratification system

**Figure 4:**
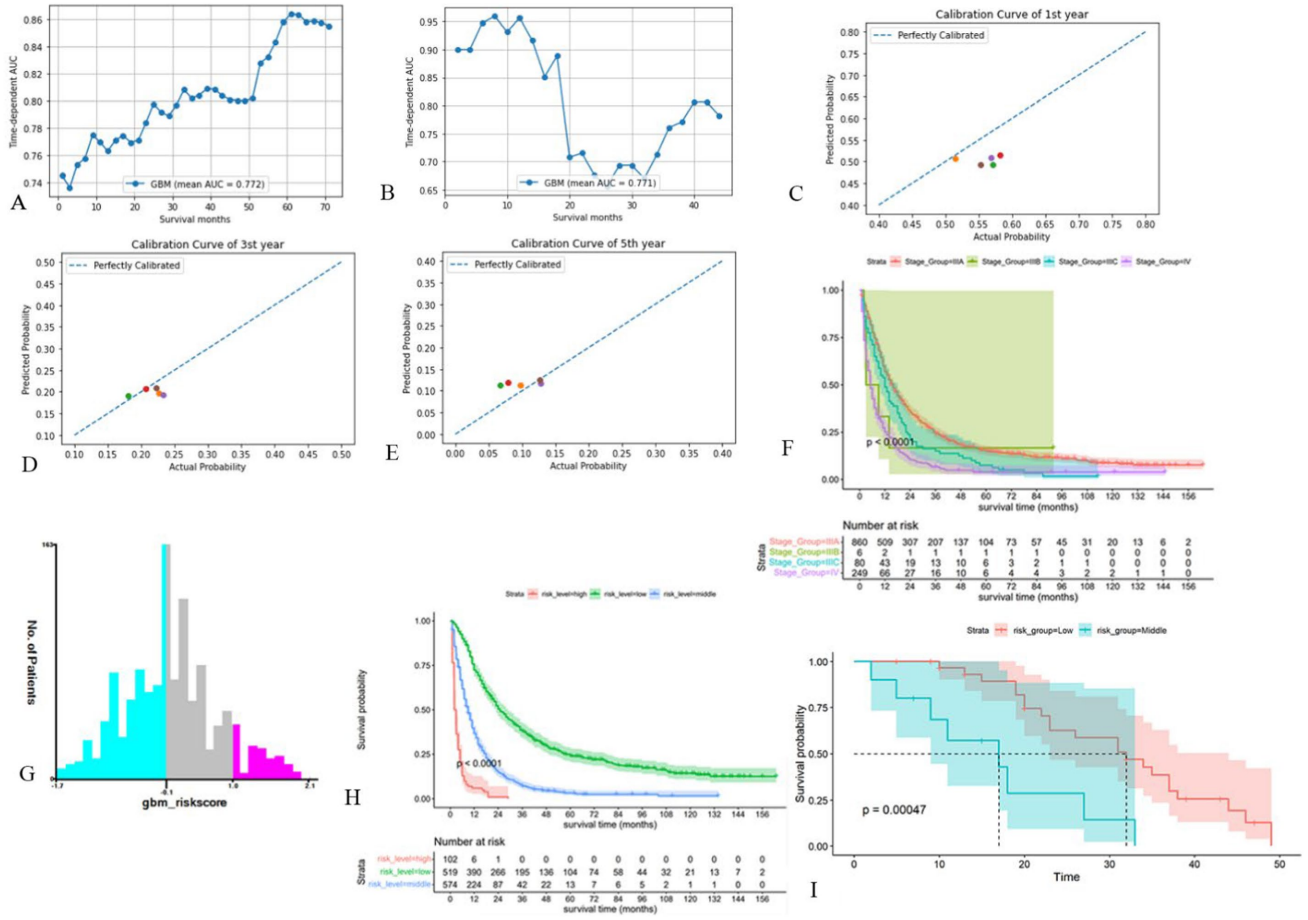
Validation of the GBM model and development of new risk stratification system. (**A, B**) Time-dependent AUC for the GBM model in internal test cohort (**A**) and external test cohort (**B**). (**C-E**) Calibration curves of first (**C**), third (**D**) and fifth (**E**) year in the internal test cohort. (**F**) Survival curves based on AJCC-TNM stage. (**G**) Cut off values for optimal grouping determined using X-tile. (**H**) K-M survival curves based on new risk stratification system. (**I**) K-M survival curves of external test cohort based on new risk stratification system (Only one of these patients was high risk and was merged into the intermediate risk group).

We performed internal and external tests. Internal and external test cohorts consisted of 512 and 41 patients, respectively. The mean AUC values of GBM model over the period from the 1st to the 72nd month was 0.772 and increased over time (Figure 4A). In external cohort, the average AUC value was 0.771, which is surprisingly high in the first year (Figure 4B) its C-index was 0.702 and Brier score was 0.129 in internal test cohort, with C-index of 0.691 and Brier score of 0.136 in external test cohort (Supplementary Table S4). Calibration curves revealed that the model’s predictions were highly consistent with the actual situation (Figures 4C–E). Therefore, the GBM model still performed well in the test cohort. Figure 4F showed the poor ability of TNM stage to differentiate patients’ prognosis (Figure 4F), to assess the model’s ability to differentiate patients’ OS, we developed a risk stratification system based on the total risk score of each patient in the training cohort and determined the optimal cut off value using X-tile software (Figure 4G). Patient risk scores were determined from the GBM model’s predictions and they ranged from between −1.7 and 2.1, with lower than −0.1 being low risk, higher than 1.0 being high risk, and in between being intermediate risk. Following this, we plotted the K-M survival curves for the three risk subgroups (Figure 4H), which showed significant differences in prognosis among the different subgroups, with the high-risk group having the worst prognosis and the low-risk group having a better prognosis. The prognosis of external test cohort was similarly well differentiated (Figure 4I).

### 3.5 Interpretation of GBM model and feature importance

**Figure 5:**
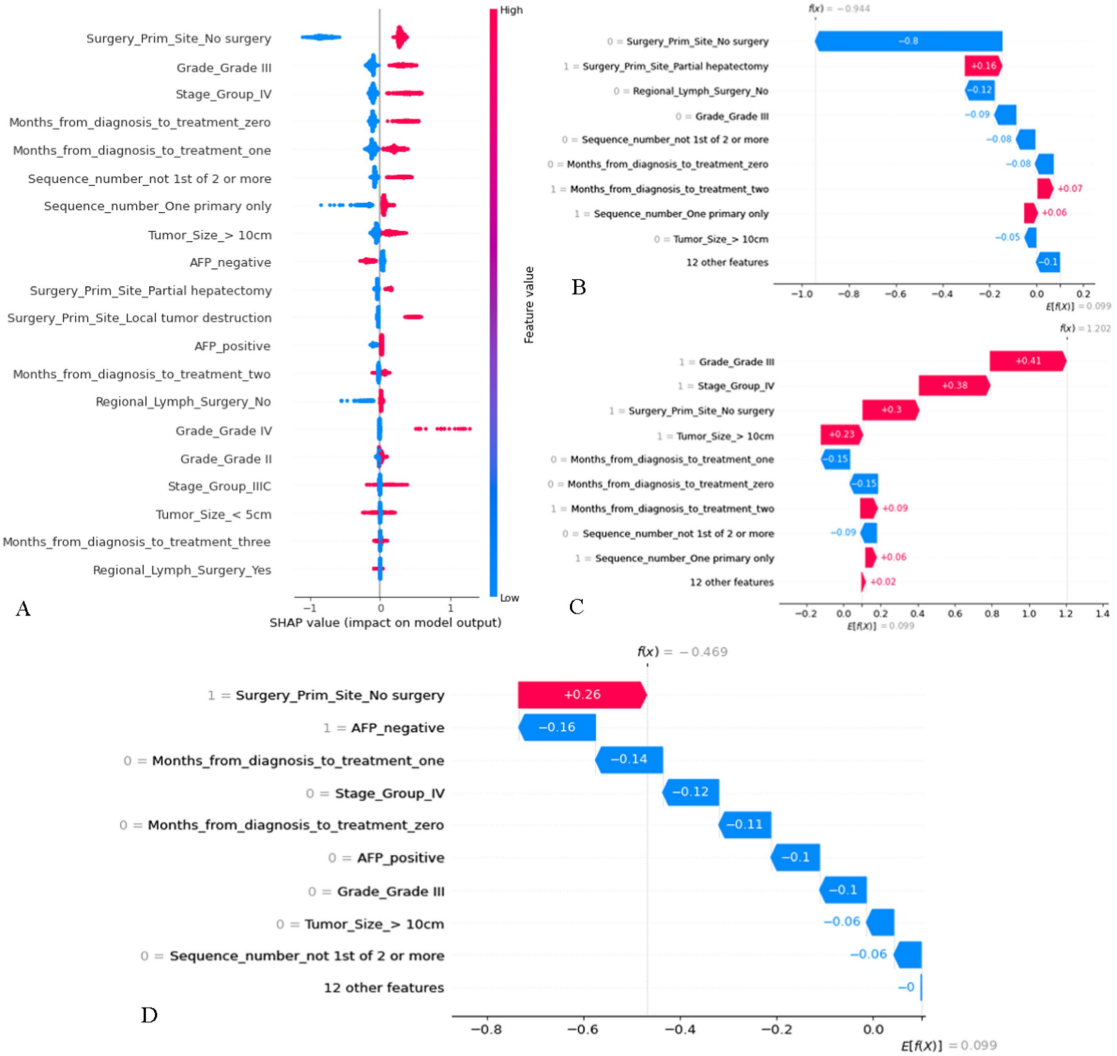
The SHAP plot of the GBM model. (**A**) SHAP beeswarm summary plot on the impact of input variables on the GBM model’s prediction. (**B**) The local SHAP plot of patient #1. Patient #1: 74-year-old male, survival time was 96 months, alive. AJCC TNM stage was IIIA, Histological grade was II, tumor size = 6.0 cm, AFP was positive. She was treated 2 months after diagnosis, underwent partial hepatectomy and regional lymph surgery, only had HCC in his life. (**C**) The local SHAP plot of patient #2. Patient #2: 42-year-old male, survival time was 2 months, died. AJCC TNM stage was IV, Histological grade was III, tumor size = 13.0 cm, AFP was negative. She was treated 2 months after diagnosis, no tumor site and regional lymph surgery, only had HCC in his life. (**D**) The local SHAP plot of patient #3. Patient #3: 82-year-old male, survival time was 7 months, died. AJCC TNM stage was IIIA, Histological grade was II, tumor size = 5.9 cm, AFP was negative. She was treated 1 month after diagnosis, no tumor site and regional lymph surgery. Only had HCC in his life. The red ribbons in the local SHAP plot represent risk factors that lead to a poor prognosis, whereas the blue ribbons are the relatively protective factors.

Features with higher mean Shapley values are more important for prognosis, and in the SHAP plot (Figure 5A), the features were listed in descending order of importance. Among them, whether the tumor primary site was operated on was the most important. In addition, a positive SHAP value increases the probability of death, i.e., the higher the value, the higher the risk of death, and vice versa. The results suggested that histological grade of grade III and TNM stage of stage IV increased the probability of death. As for “tumor primary site surgery,” no surgery generally increased the probability of death, but it is not difficult to find that in a considerable number of cases, no surgery would increase the probability of survival. Three patients from the training cohort were selected for the prognostic demonstration (Figures 5B–D). The first patient underwent partial hepatectomy, which increased the probability of death, while the next two patients had the opposite effect, with an increase in the probability of death due to no surgical intervention. Therefore, many patients with advanced MHCC may have lost the opportunity for surgery at the time of diagnosis, and it is necessary to strictly grasp the indications for surgery in order to make the patients benefit from surgery. To facilitate the use of our prognostic model by clinicians, we built a website,^1^ which allows users to directly input their own data for prediction of OS and risk score. Controlling for the same other features and then inputting a different treatment to determine if the prediction improves or decreases, by which they can also preliminarily assess whether a treatment is beneficial.

## 4 Discussion

The morbidity and mortality rates of HCC are increasing annually, and the treatment of MHCC is more complicated than that of solitary HCC, and once it reaches an advanced stage, the prognosis of the patient is quite dismal (21). Importantly, clinicians need to balance commonly used treatments at this stage, and there is a lack of effective predictive models to the extent that some patients are not treated rationally enough (22). Our research is an attempt to build predictive models for advanced MHCC patients using well-established Cox regression, ML and DL algorithms.

Our results indicated that the GBM model had the best prediction accuracy with a C-index of 0.730 and a Brier score of 0.111, and the AUCs for the 1st year, 3rd year, and 5th year were higher than 0.78 with an increasing trend. In addition, the GBM model still performed well in the test cohort, which demonstrated that our model is quite reliable in terms of prediction accuracy. The DCA curves indicated that the use of our GBM model maximized the survival benefit for patients with advanced MHCC. DeepSurv model uses a DL neural network to integrate Cox proportional hazards, which performed slightly weaker with the GBM model in this study.

According to Cox regression analysis, our model included 8 variables, which were shown in Table 1. The higher the histological grade, the worse the differentiation, the later the TNM stage, and the worse the OS of HCC patients, which has been recognized by the public. AFP is currently the most commonly used tumor marker for HCC, and according to the Asian HCC guidelines, the serum biomarker AFP is recommended as one of the monitoring and diagnostic tools for HCC (23, 24), however, many non-cancer sources involving liver and other organs may also lead to elevated AFP and thus have lower sensitivity and specificity (25). Limited literatures addressed the clinical significance of regional lymph node dissection during surgery in patients with HCC, a study by Yang et al. based on the SEER database reported that regional lymph node dissection was not an independent prognostic factor for OS (26). Another report showed a significantly higher incidence of postoperative ascites and a significantly lower overall tumor recurrence rate for liver surgery combined with regional lymph node dissection versus no lymph node dissection, although there was no difference in OS rates (27). The clinical significance of regional lymph node dissection in advanced MHCC remains to be studied. As for surgery for primary tumor sites, multivariate Cox results demonstrated that liver surgery improved OS for patients with advanced MHCC in general, and liver transplantation in particular. However, the subsequent SHAP figure indicated that a considerable number of patients with advanced MHCC were not suitable for surgical treatment, and no surgery was a kind of protection. Although a number of studies on MHCC have shown that hepatectomy (28, 29), LT, and even combined ablation therapy were effective treatment strategies for MHCC (30), Bartolini et al. (11) reported that surgery should be subject to strict indications in order to benefit specific patients, especially for patients with advanced MHCC. Our model may help clinicians make decisions, but further test is needed. For solitary HCC, tumor size often affects treatment and prognosis (31), but in our study, for advanced MHCC, there did not appear to be a significant difference in risk of death between different tumor sizes.

Interestingly, sequence of malignant tumors and interval from diagnosis to treatment showed results that seemed to differ from popular perception. Patients with HCC alone had worse OS than those who developed other primary tumors after HCC, and we found that other researchers have reported similar results (32, 33). They noted that patients with only one cancer may die prematurely due to poor health or a higher degree of malignancy of the tumor, with no chance of getting other tumors, and that re-emergence of other tumors occurs only in patients who have been survival for a long time. Secondly, patients with HCC only may have defective immune surveillance, leading to “immune escape,” while reoccurrence of other tumors may activate cancer-related immune mechanisms. Finally, patients who re-emerge with other tumors will inevitably receive additional anti-tumor treatments, and these subsequent treatments may act as concurrent anti-HCC therapies. There is no uniformity in the literature regarding the impact of the time interval between diagnosis and treatment on prognosis. One study reported that time delay from diagnosis to treatment did not significantly affect OS in HCC patients (34), but Tsai et al. reported that the longer the time interval between diagnosis and treatment of early liver cancer, the lower the OS was (35). Therefore, randomized controlled trials may be needed to clarify the clinical significance of this factor.

Our research is advanced. We first applied multiple algorithms to construct prognostic models for patients with advanced MHCC, which were evaluated by multiple methods, and ultimately the more superior GBM model was selected among five models. To our knowledge, this is the first model for advanced MHCC. Visualization and application promotion of ML models are difficult problems, we used SHAP technique for model interpretation and built a prediction website to solve this problem well. Despite a substantial amount of published research indicating that AI-based systems demonstrate significant advantages in improving the accuracy and efficiency of HCC screening, diagnosis, and tumor characterization, there is still a need for rigorous multicenter prospective validation studies and the validation of standardized multimodal datasets (36, 37). Secondly, the SEER database only covers cancer data in the U.S. Our study would be more convincing if more data were obtained. Due to the limitations of the SEER database, some variables that may be important, such as BCLC stage, genetic factors and targeted therapies, are not available. Having access to these variables may improve the performance of the model.

## 5 Conclusion

A total of eight variables were independent prognostic factors, which were included in the model to predict the prognosis of patients with advanced MHCC, and the GBM model could provide a more accurate prediction of patients’ OS.

## Data Availability

Publicly available datasets were analyzed in this study. This data can be found at: the Surveillance, Epidemiology, and End Results (SEER) database (https://seer.cancer.gov/mortality/).

## Footnotes

^1^http://39.101.130.191:8888/mhcc
