# Supplementary material for: Development of prognostic models for advanced multiple hepatocellular carcinoma based on Cox regression, deep learning and machine learning algorithms
Source: Front Med (Lausanne). 2024 Sep 27;11:1452188. doi: 10.3389/fmed.2024.1452188 (PMC11466773; doi:10.3389/fmed.2024.1452188)
Supplement: Supplementary file 2 [file Data_Sheet_2.docx]

Supplementary Material

**Table S1.** Univariate cox regression analysis of training cohort.

| **Characteristics** | **HR** | **95%CI** | **P Value** | |
| --- | --- | --- | --- | --- |
| Sex |  |  |  | |
| Female | reference |  | |  |
| Male | 1.28 | 1.09-1.49 | | < 0.01 |
| TNM Stage |  |  |  |  |
| IIIA | reference |  | |  |
| IIIB | 1.45 | 0.60-3.49 | | 0.41 |
| IIIC | 1.40 | 1.11-1.76 | | < 0.01 |
| IV | 2.29 | 1.97-2.65 | | < 0.001 |
| Histological grade |  |  | |  |
| Grade I | reference |  | |  |
| Grade II | 0.93 | 0.81-1.08 | | 0.36 |
| Grade III | 1.48 | 1.25-1.75 | | < 0.001 |
| Grade IV | 1.53 | 0.95-2.47 | | 0.08 |
| Surgery |  |  | |  |
| No surgery | reference |  | |  |
| Local tumor destruction | 0.54 | 0.42-0.69 | | < 0.001 |
| Partial hepatectomy | 0.39 | 0.33-0.45 | | < 0.001 |
| Liver transplantation | 0.14 | 0.09-0.24 | | < 0.001 |
| Regional lymph surgery |  |  | |  |
| No | reference |  | |  |
| Biopsy | 0.81 | 0.37-1.71 | | 0.59 |
| Yes | 0.49 | 0.37-0.64 | | < 0.001 |
| Sequence of systemic and surgery |  |  | |  |
| No systemic treatment and/or surgery | reference |  | |  |
| before surgery | 0.28 | 0.20-0.41 | | 0.001 |
| after surgery | 0.75 | 0.58-0.96 | | < 0.05 |
| both before and after | 0.57 | 0.27-1.19 | | 0.14 |
| Intraoperative | 0.84 | 0.51-1.37 | | 0.48 |
| Tumor size |  |  | |  |
| < 5cm | reference |  | |  |
| 5 ~ 10 cm | 0.76 | 0.61-0.94 | | 0.56 |
| > 10cm | 0.93 | 0.74-1.18 | | < 0.05 |
| Chemotherapy |  |  | |  |
| No/Unknown | reference |  | |  |
| Yes | 1.55 | 1.35-1.77 | | < 0.001 |
| Sequence number |  |  | |  |
| One primary only | reference |  | |  |
| 1st of 2 or more | 0.38 | 0.35-0.72 | | < 0.001 |
| not 1st of 2 or more | 0.1.10 | 0.93-1.30 | | 0.25 |
| AFP |  |  | |  |
| negative | reference |  | |  |
| borderline | 1.55 | 0.64-3.75 | | 0.34 |
| positive | 1.48 | 1.28-1.71 | | < 0.001 |
| Months from diagnosis to treatment |  |  | |  |
| 4 or more | reference |  | |  |
| zero | 1.64 | 1.31-2.07 | | < 0.001 |
| one | 1.59 | 1.30-1.94 | | < 0.001 |
| two | 1.25 | 1.01-1.54 | | < 0.05 |
| three | 1.16 | 0.88-1.42 | | 0.37 |

**Table S2.** C-index and Brier score of five prognostic models.

|  | **C-index** | **Brier score** |
| --- | --- | --- |
| CPH | 0.713 | 0.117 |
| Tree | 0.639 | 0.130 |
| RSF | 0.689 | 0.127 |
| GBM | 0.730 | 0.111 |
| DeepSurv | 0.714 | 0.107 |
| Random | 0.500 | 0.250 |

**Table S3.** Specific parameters of five prediction models.

| **Model** | **parameter** | **value** |
| --- | --- | --- |
| CPH | all | default |
| Tree ^a^ | max_depth | 9 |
|  | min_samples_split | 2 |
|  | min_samples_leaf | 20 |
|  | min_weight_fraction_leaf | 0.2 |
|  | max_features | 8 |
|  | max_leaf_nodes | 4 |
|  | Others ^b^ | default |
| RSF | n_estimators | 180 |
|  | max_depth | 3 |
|  | min_samples_split | 2 |
|  | min_samples_leaf | 14 |
|  | max_features | 4 |
|  | Others | default |
| GBM | subsample | 0.89 |
|  | min_samples_leaf | 5 |
|  | Others | default |
| DeepSurv | dropout | 0.2 |
|  | batch_size | 220 |
|  | learn_rate | 0.001 |
|  | epochs | 100 |
|  | Others | default |

^a^ “Tree” refers to survival tree model.

^b^ “Others” refers to other parameters not mentioned in the table and took default values.

**Table S4.** **The GBM model’s performance in internal test cohort and external test cohort.**

| **Model** | **Internal test cohort** | | **External test cohort** | |
| --- | --- | --- | --- | --- |
|  | **C-index** | **Brier Score** | **C-index** | **Brier Score** |
| GBM model | 0.702 | 0.129 | 0.691 | 0.136 |
| Random | 0.500 | 0.249 | 0.500 | 0.214 |


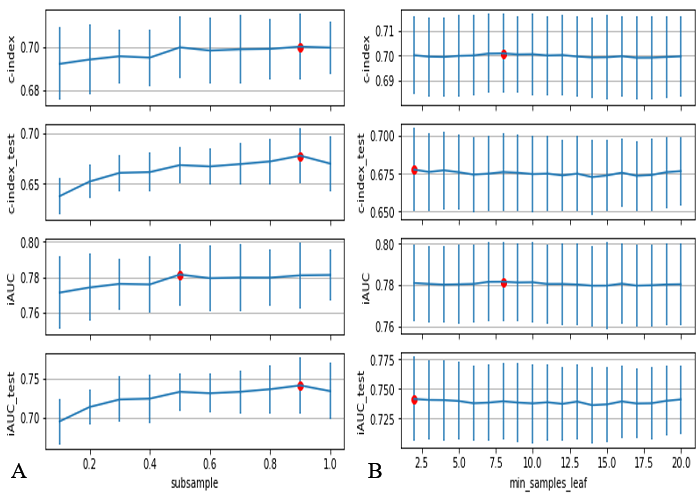


**Figure S1.** Parameter optimization of GBM model with five-fold cross-validation. (**A**) Visualization of grid search for parameters “n_subsample”. (**B**) Visualization of grid search for parameters “min_samples_leaf”.
